# Supplementary material for: Donor Screening Revisions of Fecal Microbiota Transplantation in Patients with Ulcerative Colitis
Source: J Clin Med. 2022 Feb 17;11(4):1055. doi: 10.3390/jcm11041055 (PMC8879222; doi:10.3390/jcm11041055)
Supplement: Supplementary file 1 [file jcm-11-01055-s001.zip › jcm-1540109-supplementary.pdf]

Supplemental Table S1. Reasons for failed laboratory testing (5/2018–9/2021).

Test item / No.[1]	Fecal testing					Blood testing			
	Fecal occult blood testing	*Clostridioides difficile*-specific GDH[2]	*Escherichia coli*	Other enteric pathogens	Parasites	Renal function	Liver function	Epstein-Barr virus Ig[4]M	CRP[3]
1							+		
2					+				
3	+								
4					+				
5					+				
6								+	
7					+				
8				+	+				
9						+			
10					+				
11					+				
12					+	+			
13							+		
14					+				
15		+			+				
16	+				+				
17			+						
18							+		
19			+				+		+
20							+	+	
21			+				+		
22			+		+				
23			+						
24					+				
25			+	+	+				
26					+				
27			+						
28			+						
29					+				
30					+				
31									+
32				+					
33			+						
34					+				
35					+				
36			+						
37			+		+		+		
Sample No.	2	1	11	3	20	2	7	2	2

[1]No., number; [2]GDH, glutamate dehydrogenase; [3]CRP, C-reactive protein; [4]Ig, immunoglobulin. The patient numbers are in chronological order.

Supplemental Table S2. Result of parasite-specific antibody screening testing.

Parasite / No.[1]	*Dirofilaria immitis*	*Ascaris suum*	*Anisakis*	*Gnathostoma spinigerum*	*Cysticercus cellulosae*	*Strongyloides stercoralis*	*Paragonimus westermanii*	*Clonorchis sinensis*	*Sparganum mansoni*
2						**1+**			
4						**1+**		**1+**	
5						**1+**			
7						**1+**			
8						**1+**			
10				**1+**		**1+**			
11				**1+**		**1+**			
12						**1+**			
14		**1+**	**1+**	**1+**	**1+**				
15			**1+**			**1+**			
16						**1+**			
22				**1+**		**1+**			
24				**1+**					
25				**1+**					

26									**1+**
29		**1+**					**1+**	**1+**	
30				**1+**					
34	**1+**								
35								**1+**	
37								**1+**	
Sample No.	1	2	2	7	1	11	1	4	1

The patient number is the same as the number in Supplement table 1. [1]No., number. Parasite antibody positive degree: 0, negative; 1+, false positive; 2+, weakly positive; 3+, positive; 4+, strong positive.
